# Systemic Administration of the TRPV3 Ion Channel Agonist Carvacrol Induces Hypothermia in Conscious Rodents

**DOI:** 10.1371/journal.pone.0141994

**Published:** 2015-11-03

**Authors:** Viktor V. Feketa, Sean P. Marrelli

**Affiliations:** 1 Department of Molecular Physiology and Biophysics Graduate Program, Cardiovascular Sciences Track, Baylor College of Medicine, Houston, Texas, United States of America; 2 Department of Anesthesiology, Baylor College of Medicine, Houston, Texas, United States of America; Federico II University of Naples, ITALY

## Abstract

Therapeutic hypothermia is a promising new strategy for neuroprotection. However, the methods for safe and effective hypothermia induction in conscious patients are lacking. The current study explored the Transient Receptor Potential Vanilloid 3 (TRPV3) channel activation by the agonist carvacrol as a potential hypothermic strategy. It was found that carvacrol lowers core temperature after intraperitoneal and intravenous administration in mice and rats. However, the hypothermic effect at safe doses was modest, while higher intravenous doses of carvacrol induced a pronounced drop in blood pressure and substantial toxicity. Experiments on the mechanism of the hypothermic effect in mice revealed that it was associated with a decrease in whole-body heat generation, but not with a change in cold-seeking behaviors. In addition, the hypothermic effect was lost at cold ambient temperature. Our findings suggest that although TRPV3 agonism induces hypothermia in rodents, it may have a limited potential as a novel pharmacological method for induction of hypothermia in conscious patients due to suboptimal effectiveness and high toxicity.

## Introduction

Therapeutic hypothermia (TH), defined as an intentional mild lowering of the patient’s core temperature, is emerging as a remarkably effective method of neuroprotection from ischemia [1]. It has demonstrated a benefit for survival and functional outcome in clinical trials in anesthetized patients resuscitated after out-of-hospital cardiac arrest [2,3] and is also being explored for the treatment of stroke, traumatic brain injury and other diseases [4–7]. However, induction of hypothermia in mammals remains a challenging problem because their thermoregulatory system attempts to maintain a stable level of body temperature. The current methods of hypothermia induction rely on the combination of physical cooling by surface or endovascular devices with suppression of the thermoregulatory responses by anesthetic, sedative and muscle paralytic drugs [8]. Because of the requirements for complicated equipment, procedures and intensive monitoring, the application of hypothermia is limited only to the most specialized care settings. Moreover, the use of aggressive anti-shivering drug regimens has a high potential for dangerous side effects. Thus, novel safe and effective methods of hypothermia induction are needed to expand the indications and promote clinical adoption of TH.

A promising experimental alternative to current methods of hypothermia is the manipulation of the afferent neural pathways for peripheral temperature sensation, which allows controlling the effector activities of the thermoregulatory system to produce a drop in core temperature [8]. Put in simpler metaphorical terms, this approach is based on pharmacologically tricking the thermoregulatory system into sensing heat and lowering core temperature as a defense against perceived overheating. A strikingly effective example of this strategy is provided by pharmacological activation of the Transient Receptor Potential cation channel, subfamily V, member 1 (TRPV1), also known as the heat and capsaicin receptor, which is located within the skin-warming afferent neural pathways [9–11]. TRPV1 activation by parenterally administered agonists effectively lowers core temperature in numerous mammalian species, likely by sending spurious information to the hypothalamus about extreme peripheral warming and inducing the net heat loss state of the thermoregulatory system [8,11–13]. However, TRPV1 agonists may also activate TRPV1-expressing nociceptive neurons and induce a conscious perception of burning pain [14]. The potential for this side effect hinders further exploration of TRPV1 agonists as hypothermic agents in human patients. Thus, it is desirable to discover additional pharmacological targets for controlling the afferent pathways for temperature sensation that would lack the adverse side effects related to TRPV1.

To this end, the current study focused on the TRPV3 ion channel, which is closely related to TRPV1 and was also implicated in thermosensation [15]. Unlike TRPV1 channels, which are expressed predominantly in primary sensory neurons of the dorsal root ganglion, TRPV3 is mainly expressed in skin keratinocytes [16]. This localization initially cast doubt on the involvement of TRPV3 in temperature sensing. However, a study in TRPV3 knock-out animals provided strong evidence that TRPV3 is indeed required for proper sensing of the warm temperatures in the mild and noxious range [17]. Another important difference between TRPV1 and TRPV3 is the activation temperature *in vitro*: while TRPV1 is activated by temperatures above 42°C, which corresponds to the psychophysical sensation of burning heat, TRPV3 is activated above 33°C, corresponding to the feeling of mild warmth [17]. On one hand, these properties of TRPV3 suggest that its pharmacological activation also induces a conscious perception of pleasant warmth, in contrast to the burning pain sensation induced by TRPV1 activation. On the other hand, TRPV3 activation may be expected to provide the afferent signal about skin warming to the autonomic thermoregulatory centers in the hypothalamus and, similarly to TRPV1 activation, induce an autonomically mediated net heat loss state and a drop in core temperature. However, this prediction has not been yet directly tested. Therefore, this study aimed to test the hypothesis that TRPV3 activation by the systemically delivered agonist carvacrol produces a drop in core temperature in conscious freely-behaving rodents, as well as to identify the possible mechanisms of the putative hypothermic effect of TRPV3 activation by carvacrol.

## Materials and Methods

### Animals

All animal experiments were approved by the Institutional Animal Care and Use Committee at Baylor College of Medicine. Mice and rats were used for this study. Mice were of C57Bl/6 or CD-1 strain, ~10 weeks of age, male, and weighed 20–40 g. Sprague-Dawley rats were 13–22 weeks of age, male, and weighed 250–500 g. Animals were housed in the dedicated animal facility, in groups of 2–5 mice or 2–3 rats per cage, at an ambient temperature of ~24°C, with 12 h light/dark cycle and *ad libitum* access to water and standard chow. During experiments, animals were housed individually.

### Drugs

To activate the TRPV3 ion channel, the agonist carvacrol (Sigma, St. Louis, MO) was used. Carvacrol is a pungent ingredient of oregano oil and was shown to be a highly potent agonist of the TRPV3 channel in vitro [18]. Because carvacrol is poorly soluble in aqueous solutions, a 10% water solution of a detergent Solutol HS-15 (Sigma, St. Louis, MO) was selected as a solubilizing vehicle. Because carvacrol is known to be air and moisture sensitive, working solutions were freshly prepared immediately before injection by weighing the appropriate amount of carvacrol, adding to vehicle solution to obtain a 10 mg/ml initial solution, vortexing for 5 min, and diluting with vehicle to appropriate working concentration. For mouse studies, carvacrol solutions were injected intraperitoneally (IP) at 10–100 mg/kg in a volume of 10 ml/kg body weight, or intravenously (IV) at 6–100 mg/kg in a volume of 5 ml/kg body weight, using 1 ml syringes and 27G needles. For rat studies, carvacrol solutions were injected intraperitoneally at 100 mg/kg in a volume of 5 ml/kg body weight using 3 ml syringes and 25G needles, or intravenously at 25–50 mg/kg in a volume of 1.25 ml/kg body weight, using 1 ml syringes and 27G needles. It should be noted that the method of preparation of the carvacrol solutions described above appears to be very important for obtaining the hypothermic effect, because other methods of preparation used in our preliminary studies produced less consistent solubilization and effects on core temperature.

As a validation control in blood pressure measurement experiments, phenylephrine hydrochloride (Sigma, St. Louis, MO) was prepared in phosphate-buffered saline.

### Core temperature measurements

Core temperature was measured in conscious freely-behaving mice or rats by miniature wireless Implantable Programmable Temperature Transponders (IPTT-300, BioMedic Data Systems Inc., Seaford, DE). The biocompatible transponders have cylindrical shape, 14 mm in length x 2 mm in diameter. Transponders have an accuracy of ±0.5°C, resolution of 0.1°C and were factory-calibrated. Transponders were implanted subcutaneously in the back region using a manufacturer-supplied needle injector under brief isoflurane anesthesia. All experiments started at least 72 hours after transponder placement. Core temperature readings of the implanted transponders were obtained by a wireless handheld reader device (DASwr-7007S, BioMedic Data Systems Inc., Seaford, DE).

### Whole-body energy production measurements by indirect calorimetry

To determine the effect of carvacrol on energy expenditure of mice, the latter was measured by indirect calorimetry in the Comprehensive Laboratory Animal Monitoring System (“CLAMS”/Oxymax Calorimetric System, Columbus Instruments, OH). Mice were placed individually into metabolic chambers (with dimensions of 20.2cm x 10.4cm x 10 cm) with free access to food and water for the duration of the study, approximately 5 hours. Mice were on raised floors to prevent blockage of air flow into the chambers. Air flow for each chamber was set to 0.5 liters per minute. The temperature in the chamber was maintained at 25°C. The volumes of oxygen consumed and carbon dioxide expired in each individual chamber were monitored in regular intervals. After 1 hour of baseline measurements, mice were intraperitoneally injected with carvacrol or vehicle, and returned to the chambers. Measurements were continued for the next 4 hours. Core temperatures of mice in the metabolic chambers were also measured by implanted wireless temperature transmitters every 15 min throughout the protocol. Energy expenditure was determined from the gas exchange values by a conventional calculation (E = [3.815+1.232 * V(CO_2_)/V(O_2_)]*V[O_2_], where E—energy expenditure, V(O_2_)—oxygen consumption, VO_2_)—carbon dioxide production)

### Temperature preference assay

A temperature preference assay was used to determine if carvacrol affects the afferent pathways for conscious temperature perception and induces thermoregulatory behaviors. The assay was performed using a custom-built setup, which consisted of two adjacent water-jacketed steel platforms. The temperature of each platform base was independently controlled using two circulating water baths. A plastic enclosure was constructed around these two platforms, creating two contiguous surfaces (6 by 6 inches) with two different regulated temperatures, enclosed by plastic walls. One platform was maintained at 30°C (“warm”) and the other at 10°C (“cold”). 5 min after intravenous injection of carvacrol or vehicle, mice were introduced to the warm side and allowed to freely move between warm and cold sides. Mice were then recorded on video for the next 3 min, during which the time spent on each platform was measured. In the event that mice spent more than 30 seconds on either platform without venturing to the other side, they were gently nudged to the opposite side of the chamber. In this assay, a lower percentage of time spent on the cold side is thought to represent the increased sensitivity and aversion to cold. Accordingly, the loss of cold sensitivity leads to a higher percentage of time spent on the cold side.

### Ambient cold exposure

To determine the hypothermic effects of carvacrol in cold ambient temperature, mice were exposed to ambient cooling by forced cooled air in a custom-built temperature-controlled chamber. The chamber consisted of a Plexiglass enclosure (20 x 20 x 12 cm), built around a Peltier element (area = 20 x 12 cm), equipped with heat exchangers and fans (TE Technology, Traverse City, MI). The air inside the chamber was recirculated by a computer fan (~8 cm in diameter) directed over the heat exchange element and away from the mice, ensuring a uniform distribution of air temperature. The Peltier element was regulated by a temperature controller interfaced with a computer, allowing air temperature control to within 0.1°C.

### Blood pressure measurements

To evaluate the effects of carvacrol on vital physiological parameters, blood pressure was measured in anesthetized CD-1 mice in response to carvacrol administration. Mice were anesthetized with 2% isoflurane delivered through a vaporizer. Core temperature of mice was maintained at 37°C by a heating pad connected to a temperature controller. Arterial catheter for blood pressure measurements was prepared from PE-10 tubing by heat-pulling the tubing end to obtain a narrow tip with a diameter of ~1/3 of that of the original tubing. Arterial catheter was inserted into mobilized femoral artery, secured with a suture, and connected to a pressure transducer, bridge amplifier, and data acquisition system (AD Instruments, Colorado Springs, CO) to record the blood pressure signal. Another catheter made of PE-10 tubing and 27 G needle was inserted into the tail vein for intravenous delivery of drugs. Blood pressure signals were acquired by the LabChart software (AD Instruments, Colorado Springs, CO) with a 1 kHz sampling rate, processed by a smoothing algorithm with a 1-sec window and analyzed offline.

A within-subject design was used, whereby each subject was successively treated with all treatments in the following order: Solutol IP (vehicle for carvacrol); Carvacrol IP at 100 mg/kg; phosphate-buffered saline IV; phenylephrine at 50 μg/kg IV (used as a validation control for reliable measurements of changes in blood pressure); Solutol IV; Carvacrol IV at 25 mg/kg; Carvacrol IV at 50 mg/kg; Carvacrol IV at 100 mg/kg. At least 3 min of baseline pre-treatment recording and 3 min of post-treatment recording was acquired. All catheters were flushed with heparinized saline between treatments. For quantitative analysis of the effects of treatments on blood pressure, pre-treatment and post-treatment values of blood pressure were calculated as an average over 60 sec prior to treatment or 180 sec after treatment respectively, and the relative change in these values was reported. Because blood pressure levels did not recover to original baseline values after cumulative Solutol and carvacrol IV administration in a fraction of subjects, the pre-Solutol values were used as baseline measures for these treatments.

### Statistics

All results are reported as the mean ± standard error of the mean. Error bars in graphs denote standard error of the mean. Statistical differences between treatment effects were assessed by unpaired two-tailed t-test when comparing two groups and by one-way Analysis of Variance (ANOVA) followed by Student-Neuman-Keuls post-hoc pairwise test when comparing three and more groups. Time course temperature data was analyzed by 2-way Repeated Measures Analysis of Variance (RM ANOVA), with the treatment group as a fixed factor and time point as a repeated measures factor, followed by Student-Neuman-Keuls test for multiple pairwise comparisons within each time point. The change in blood pressure in response to drug administration was analyzed by the one-sample two-tailed t-test with the mean of zero set as the null hypothesis (i.e. no change in blood pressure). The differences were considered statistically significant at p < 0.05. Statistical analysis was performed in SigmaPlot Version 12 (Systat Software, San Jose, CA).

## Results

### Intraperitoneal and intravenous administration of the TRPV3 agonist carvacrol decreases core temperature in conscious mice and rats

**Fig 1:**
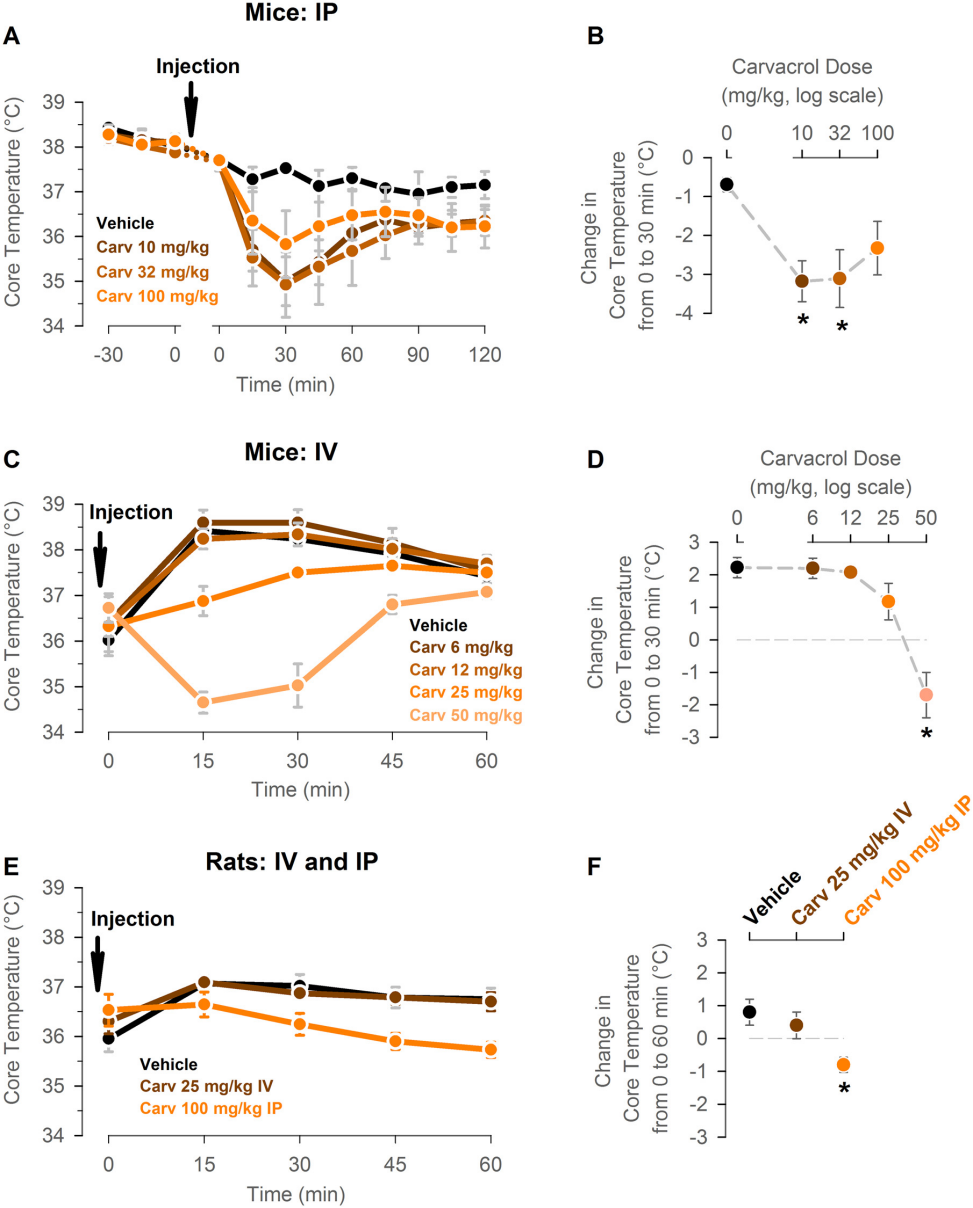
Intraperitoneal and intravenous administration of the TRPV3 agonist carvacrol decreases core temperature in conscious mice and rats. (A) Adult male C57Bl/6 mice were injected intraperitoneally with vehicle or carvacrol at 10, 31.6, and 100 mg/kg at time 0. Core temperature was measured by wireless temperature transmitters every 15 min for 2 hours. (B) The drop in core temperature between 0 min pre-injection and 30 min post-injection calculated from the experiment shown in panel A. n = 4 mice per group in a within-subject design. * P<0.05, 1-way ANOVA followed by Student-Neuman-Keuls post-hoc test. (C) Adult male CD-1 mice were injected intravenously with vehicle or carvacrol at 6, 12.5, 25, and 50 mg/kg at time 0. Core temperature was measured by wireless temperature transmitters every 15 min for 1 hour. (D) The drop in core temperature between 0 min pre-injection and 30 min post-injection was calculated from the experiment shown in panel A. n = 4–5 mice per group. * P<0.05, 1-way ANOVA followed by Student-Neuman-Keuls post-hoc test. (E) Adult male Sprague-Dawley rats were injected with vehicle of carvacrol at 25 mg/kg intravenously or 100 mg/kg intraperitoneally at time 0. Core temperature was measured by wireless temperature transmitters every 15 min for 1 hour. (F) The drop in core temperature between 0 min pre-injection and 60 min post-injection calculated from the experiment shown in panel A. n = 6–7 rats per group. * P<0.05, 1-way ANOVA followed by Student-Neuman-Keuls post-hoc test.

To test the hypothesis that systemic TRPV3 activation induces hypothermia, core temperature was measured by wireless transmitters in adult male C57Bl/6 mice before and after intraperitoneal injection with vehicle or carvacrol at different doses. Core temperature was measured every 15 min for 2 hours. Intraperitoneal injection of vehicle led to ~0.7±0.2°C drop in core temperature at 30 min after injection. Intraperitoneal injection of carvacrol at 10 and 31.6 mg/kg produced a larger drop in core temperature than vehicle injection did, while the effect of carvacrol injection at 100 mg/kg was not different from the vehicle effect (10 mg/kg: -3.2±0.5°C; 31.6 mg/kg: -3.1±0.7°C.; 100 mg/kg: -2.3±0.7°C; p<0.05 for 10 and 31.6 mg/kg, 1-way ANOVA followed by Student-Neuman-Keuls post-hoc test; n = 4 mice per group in a within-subject design; Fig 1A and 1B).

To confirm that the hypothermic effect of carvacrol is replicated with a different route of systemic administration and in a different mouse strain, core temperature was measured in adult male CD-1 mice after intravenous injection of carvacrol at 6, 12.5, 25, and 50 mg/kg. Intravenous injection of vehicle and carvacrol at 6, 12.5 and 25 mg/kg led to an increase in core temperature of mice of 2.2±0.2°C, 2.2±0.3°C, 2.0±0.2°C, and 1.2±0.6°C respectively at 30 min post-injection, and this change in temperature was not statistically different between treatments. In contrast, intravenous injection of carvacrol at 50 mg/kg led to a decrease in core temperature of -2.1±0.4°C (p<0.05 for a change in core temperature vs. veh, 1-way ANOVA followed by Student-Neuman-Keuls post-hoc test; n = 4–5 mice per group; Fig 1C and 1D). Intravenous injection of carvacrol at 100 mg/kg resulted in death of 3 out of 5 tested mice during injection, while the two surviving mice had an average drop in core temperature of 3.9±1.7°C (data for this group is not shown and was excluded from analysis).

To test if the hypothermic effect of carvacrol may be generalized to species other than mice with more robust thermoregulatory systems, the carvacrol-induced drop in core temperature was determined in conscious rats within an analogous protocol. Rats were injected with vehicle or carvacrol at 25–50 mg/kg intravenously or at 100 mg/kg intraperitoneally, and their core temperature was measured every 15 min for 1 hour. Carvacrol at 25 mg/kg IV produced a slight increase in core temperature of rats at 60 min post-injection compared with pre-injection levels, and this relative change in temperature was not different from that in vehicle-treated rats. However, carvacrol at 100 mg/kg IP caused a slight decrease in core temperature, and this change was statistically different from the effect in vehicle-treated group (vehicle: 0.8±0.4°C; carvacrol at 25 mg/kg IV: 0.4±0.4°C; carvacrol at 100 mg/kg IP: -0.8±0.2°C; p<0.05 for carvacrol at 100 mg/kg IP vs carvacrol at 25 mg/kg IV and vehicle groups, 1-way ANOVA followed by Student-Neuman-Keuls post-hoc test; n = 6–7 rats per group; Fig 1E and 1F). Intravenous injection of carvacrol at 50 mg/kg resulted in death of 3 out of 3 tested rats during injection (data for this group is not shown and was excluded from analysis).

### Carvacrol-induced hypothermia is associated with a decrease in whole-body energy production

**Fig 2:**
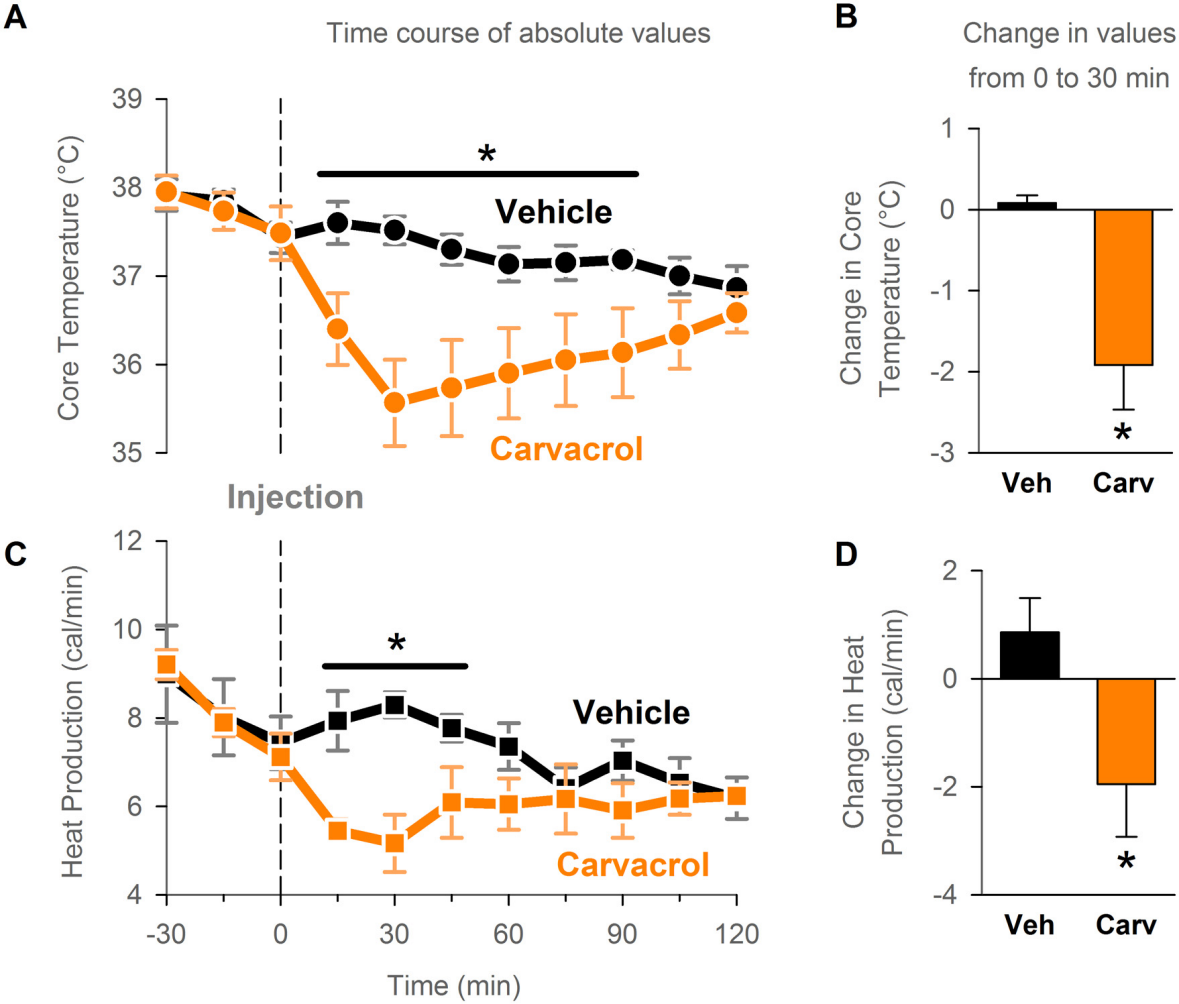
Carvacrol-induced hypothermia is associated with a decrease in whole-body energy production. Whole-body heat production was calculated from oxygen consumption (indirect calorimetry) measured in a metabolic chamber. Mice were injected intraperitoneally with vehicle or carvacrol at 31.6 mg/kg at time 0. N = 6 mice per group in a within-subject design. (A) and (C) Time course of core temperature (A) and heat production measurements (C). * P<0.05 at respective time points, 2-way Repeated Measures ANOVA/Holm-Sidak post-hoc test. (B) and (D) Change in core temperature (B) and heat production (D) from 0 min pre-injection to 30 min post-injection. * P<0.05, t-test.

Next, the possible thermoeffector mechanisms for the hypothermic effect of carvacrol in mice were explored. Hypothermia may be caused by either or both a decrease in heat generation or an increase in heat loss [8]. To determine if carvacrol decreases heat generation, the latter was measured in mice by indirect calorimetry after intraperitoneal administration of carvacrol (31.6 mg/kg) or vehicle. The hypothermic effect of carvacrol was reproduced in this experiment, as demonstrated by a decrease in core temperature at 30 min post-injection in the carvacrol-treated mice (-1.9±0.5°C), in contrast to no change in core temperature in the vehicle-treated mice (0.1±0.1°C; p<0.05 for the change in core temperature, t-test; n = 6 mice per group; Fig 2A and 2B). Carvacrol administration led to a decrease, while vehicle administration led to an increase in whole-body energy production at 30 min post-injection relative to pre-injection levels (a change of -1.95±0.98 cal/min in the carvacrol group vs. +0.86±0.64 cal/min in the vehicle group; p<0.05, paired t-test; n = 6 mice per group; Fig 2C and 2D). This finding indicates that TRPV3 activation by carvacrol reduces whole-body heat generation in conscious mice.

### Carvacrol does not affect temperature preference

**Fig 3:**
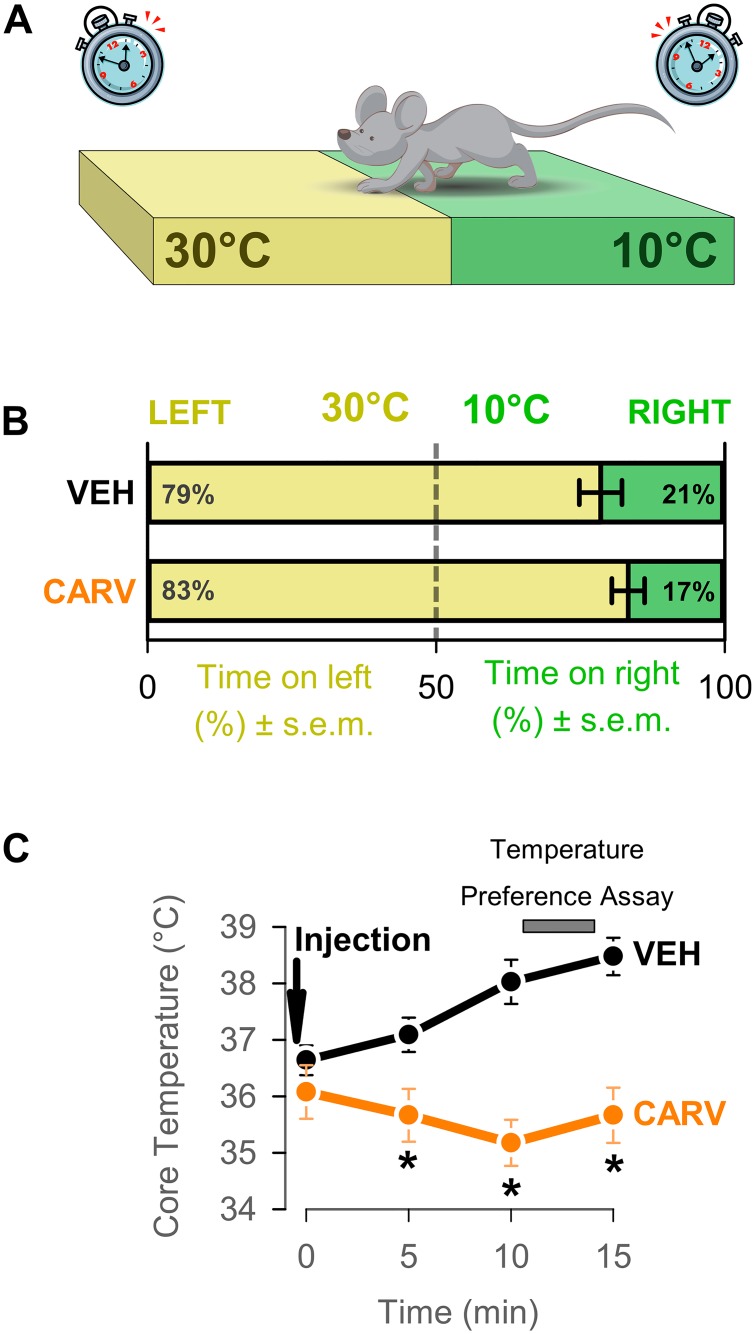
Induction of cold-seeking behaviors by carvacrol was not detected in the two-plate temperature assay. (A) Schematic of the experiment. CD-1 mice were intravenously injected with vehicle or carvacrol at 50 mg/kg. 10 min after injection, mice were introduced for 3 min into the temperature-preference apparatus, where they were allowed to move freely and select between two plates set to 30°C (left plate) and 10°C right plate). (B) The percentage of time spent on each plate was measured. This time reveals the preference for the temperature of the respective plate. N = 8 mice per group. P>0.05, t-test. (C) Core temperature was measured during the described protocol every 5 min by wireless temperature transmitters. Carvacrol injection, but not vehicle injection, induced hypothermia. * P<0.05, 2-way Repeated measures ANOVA followed by Student-Neuman-Keuls post-hoc test.

We further hypothesized that carvacrol induces hypothermia by increasing the signaling along the skin-warming afferent pathways to the hypothalamus and the cortex and inducing cold-seeking behaviors, such as a preference for cold temperature. To test this hypothesis, we determined the preference for cold temperature in the two-plate temperature preference assay after carvacrol or vehicle administration. When given the choice of residing at a plate with a surface temperature of either 30°C (thermoneutral temperature for mice) or 10°C (unpleasantly cold temperature), vehicle-treated mice preferred and spent more time on the warm side (79±4%). Preference for the warm side in mice treated with carvacrol at 50 mg/kg IV was not different from that of vehicle-treated mice (83±3%; p>0.05, t-test; n = 9 mice per group; Fig 3). These results did not support our original hypothesis and may suggest that carvacrol does not substantially activate the skin warming pathway. However, it is also possible that carvacrol-induced preference for cold temperatures may be detected when a mouse is presented with different pairs of plate temperatures, which were not tested in the current study.

### The hypothermic effect of carvacrol is lost at low ambient temperature of 10°C

**Fig 4:**
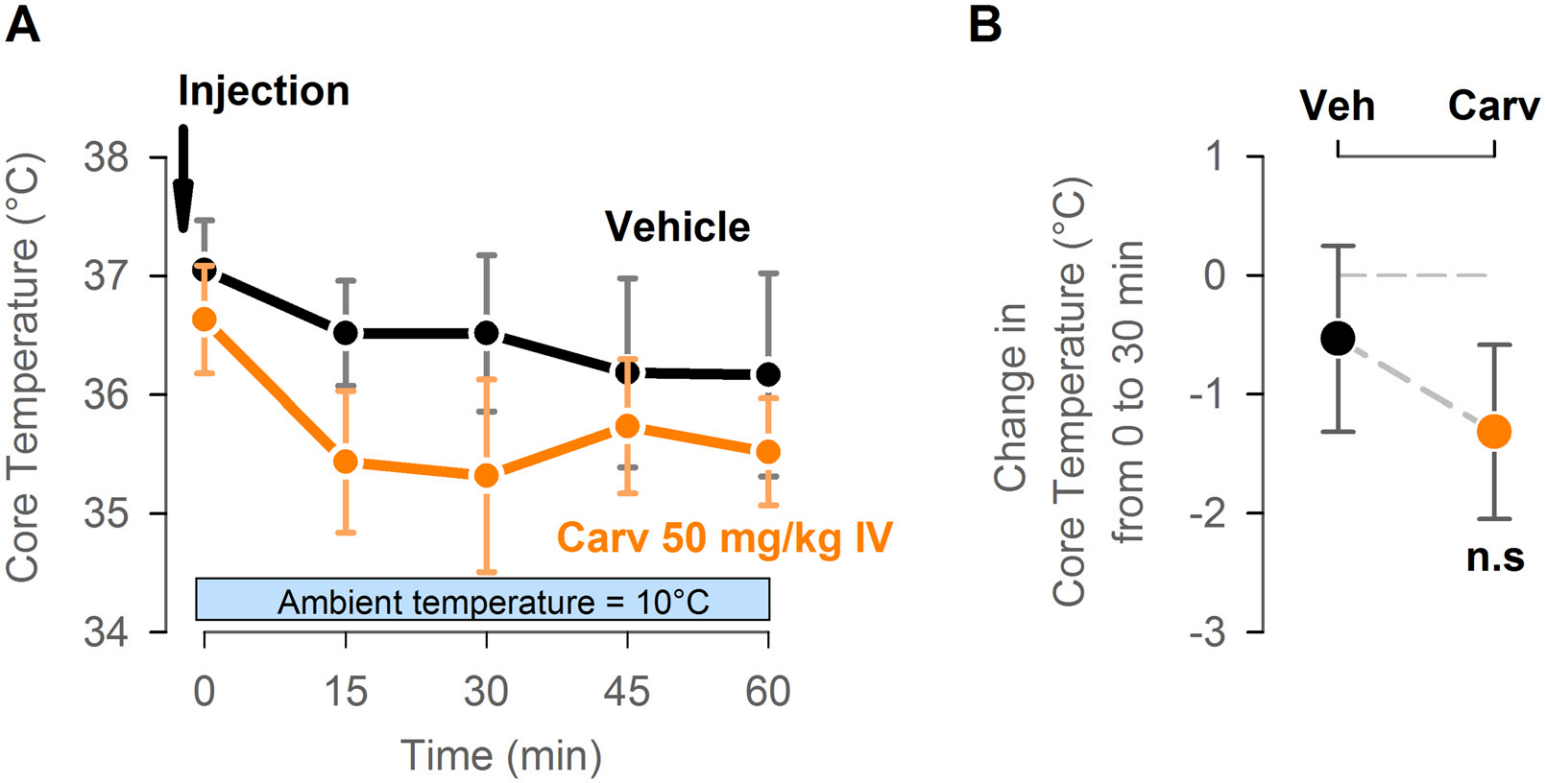
The hypothermic effect of carvacrol is lost at low ambient temperature of 10°C. (A) Adult male CD-1 mice were injected intravenously with vehicle or carvacrol at 50 mg/kg at time 0 and transferred to the temperature-controlled chamber with an ambient temperature set to 10°C. Core temperature of mice was measured by wireless temperature transmitters every 15 min for 1 hour. (B) The drop in core temperature between 0 min pre-injection and 30 min post-injection was calculated from the experiment shown in panel A. n = 6 mice per group. n.s.: P>0.05, t-test.

To determine if the hypothermic effect of carvacrol may be further enhanced when combined with conventional strategy of physical cooling, core temperature of mice was measured after intravenous injection of carvacrol at 50 mg/kg while mice were housed in a temperature controlled chamber with an ambient temperature set to 10°C. There was no statistically significant difference in change in core temperature at 30 min after injection between vehicle-treated and carvacrol-treated mice (-0.5±0.8°C and -1.3±0.7°C respectively; p>0.05, t-test; n = 6 mice per group; Fig 4). This finding suggests that carvacrol suppresses only basal heat generation, but does not suppress the compensatory cold-defensive heat generation through shivering and brown adipose tissue thermogenesis, which are triggered during exposure to cold ambient temperature.

### Carvacrol induces an acute drop in blood pressure

**Fig 5:**
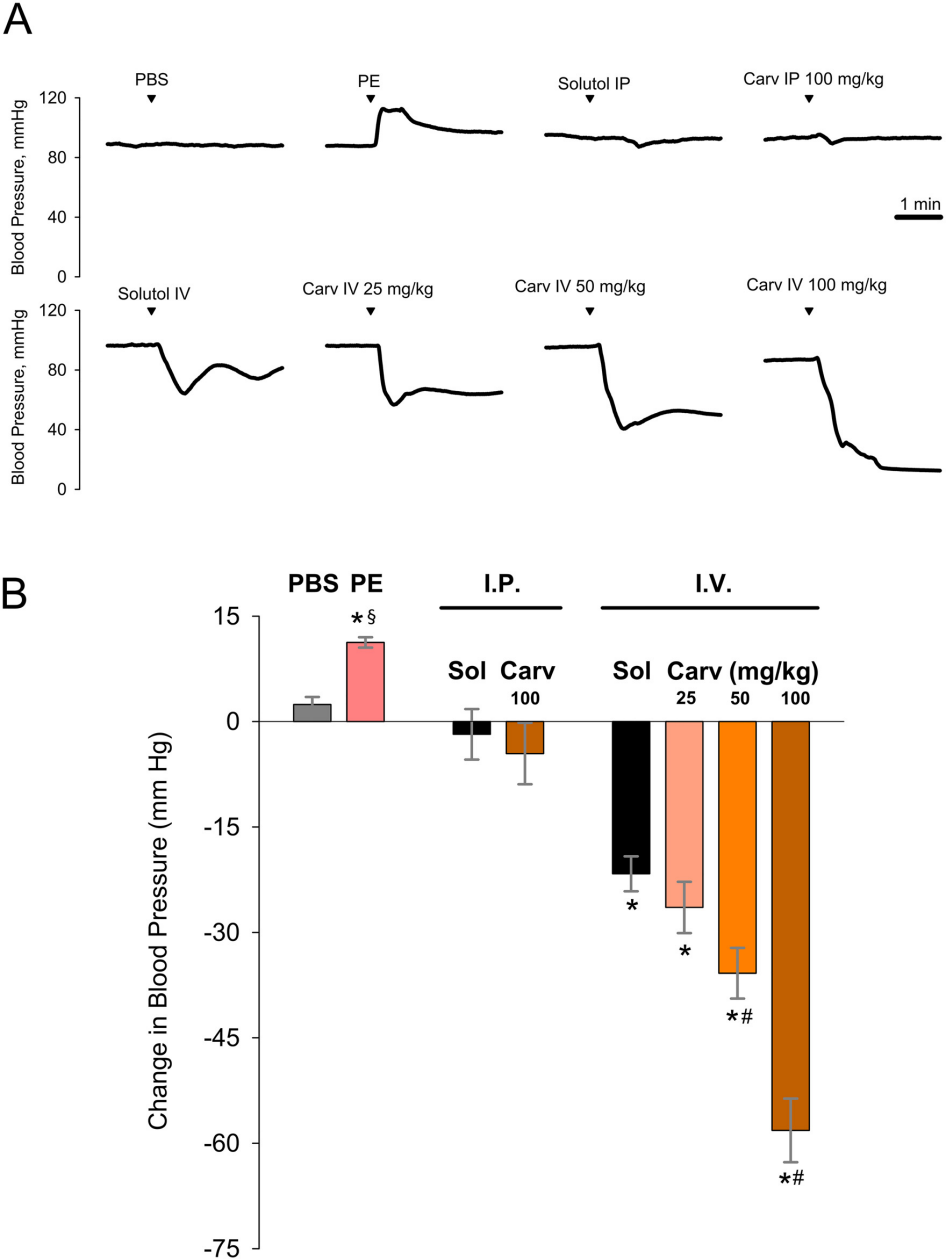
Carvacrol delivered intravenously induces an acute drop in blood pressure in anesthetized mice. (A) Representative time course graphs showing the effects of a range of doses of carvacrol delivered intraperitoneally (IP) and intravenously (IV) on blood pressure of anesthetized CD-1 mice with stably maintained normal core temperature; PBS: phosphate-buffered saline (IV), used a negative control; PE: phenylephrine, 50 μg/kg IV, used as a validation control for reliable measurements of changes in blood pressure; Carv: carvacrol. (B) The relative change between pre-treatment (calculated as an average over 1 min prior to injection) and post-treatment (an average over 3 min after injection) blood pressure levels after respective treatments. Sol.: Solutol. n = 5 mice in a within-subject design. * P<0.05, one-sample t-test with a hypothesized mean of zero as the null hypothesis (i.e. no change in blood pressure); § P<0.05 vs PBS, t test; # P<0.05 vs Solutol IP, one-way ANOVA/Student-Newman-Keuls test.

To better characterize the toxicity and lethality associated with carvacrol administration, we determined the effects of carvacrol on arterial blood pressure in anesthetized mice with stably maintained normal core temperature (Fig 5). Carvacrol injected intraperitoneally at the highest dose of 100 mg/kg, which was not associated with lethality in previous experiments, had no effect on blood pressure (change between 1-min average pre-treatment value and 3-min average post-treatment value: -5±4 mm Hg; vehicle control: -2±4 mm Hg; p>0.05, t-test; n = 5 mice per group; Fig 5B). In contrast, intravenous injections of carvacrol at 25, 50 and 100 mg/kg produced a substantial acute drop in blood pressure (-26±4, -36±4 and -58±5 mm Hg respectively), which was also associated with 100% lethality in the case of the highest dose of 100 mg/kg. Interestingly, intravenous injection of the Solutol solution, used as a vehicle for preparation of the injectable form of carvacrol, by itself produced an appreciable, albeit smaller and transient drop in blood pressure (-22±2 mm Hg; p>0.05 vs 25 mg/kg carvacrol; p<0.05 vs 50 and 100 mg/kg carvacrol; one-way ANOVA; n = 5 mice per group; Fig 5B). These findings suggest that toxic and lethal effects of carvacrol delivered intravenously may be mediated by its effect on vital cardiovascular functions, such as the maintenance of arterial blood pressure.

## Discussion

Induced therapeutic hypothermia is an effective neuroprotective strategy in some clinical contexts [8]. However, its use is limited because of the challenging logistical requirements associated with physical cooling strategies, such as the need for complicated equipment and intensive care setting, and a potential for side effects due to aggressive anti-shivering drug regimens. Wider adoption of therapeutic hypothermia may be achieved if novel methods of hypothermia induction are available that are safe, easily administered, and amenable for use in conscious subjects. The current study was aimed at identifying such novel methods based on pharmacological manipulation of the thermosensitive neural pathways. In particular, this study tested the hypothesis that systemic administration of the TRPV3 channel agonists leads to an increased skin warming signal to the autonomic center of thermoregulation in hypothalamus and induces a coordinated response to lower core temperature through inhibition of the heat-generating thermoeffector mechanisms and activation of the heat-dissipating thermoeffector mechanisms.

In partial agreement with this hypothesis, we found that the TRPV3 agonist carvacrol decreased core temperature of conscious mice after intraperitoneal and intravenous administration. However, we also observed high mortality after intravenous injection of higher doses of carvacrol, which points to its acute toxic effects. This also suggested that the hypothermic effect of carvacrol could be caused by indirect impairment of thermoregulation secondary to acute toxicity rather than by the primary and specific effect on the thermoregulatory system [19]. Core temperature of mice is known to be very sensitive to various non-specific stressors [20]. For instance, mice may experience hypothermia during fasting, hypoxia, or pathogen challenge. To determine if the hypothermic effect of carvacrol is caused by primary or secondary action on the thermoregulatory system, we attempted to reproduce this effect in rats. Thermoregulation of rats, as compared to that of mice, is more robust and their core temperature is less susceptible to non-specific decreases [21]. Thus, a decrease in core temperature of rats would be more indicative of the primary and specific thermoregulatory effect of carvacrol. We found that carvacrol injected intraperitoneally leads to a modest decrease of core temperature compared to the effect of vehicle injection. However, the hypothermic effect was not reproduced with IV administration of carvacrol at slightly lower doses. Moreover, similarly to observations in mice, higher doses of carvacrol IV led to high mortality in rats. Therefore, the hypothermic effect of carvacrol appears to be tightly linked to the general toxic effect and is much more pronounced in mice, a species highly susceptible to non-specific hypothermia. These observations argue in favor of the view that carvacrol-induced hypothermia is caused by non-specific effects on central nervous system rather than specific and safe effects on the thermoregulatory system. As a further confirmation of this view, we found that carvacrol injected intravenously produced a pronounced acute drop in arterial blood pressure independently of its temperature-lowering effects, which may be responsible for the associated toxicity and lethality.

We also sought to elucidate the thermoeffector mechanisms of the hypothermic effect of carvacrol and found that it lowers basal whole-body heat generation, as predicted by our original hypothesis. However, this finding does not allow us to differentiate whether suppression of the heat generation is a primary effect of carvacrol or secondary to its acute toxic effects.

In addition, we were not able to detect an increase in cold-seeking behavior in the two-plate temperature preference assay after carvacrol administration. This result may suggest that TRPV3 agonism activates the autonomic temperature-sensing pathways but does not affect the pathways for conscious temperature sensation. However, it is also possible that our assay was not sensitive enough to detect the activation of the cortical temperature-sensing pathways. For instance, this effect may be uncovered if other pairs of temperatures are used in the two-plate temperature preference assay. In our experiment, a mouse was offered a choice between the thermoneutral and the noxiously cold surface. Thus, any putative increase in preference for mild and pleasant cold temperature induced by carvacrol may have been masked, or overridden, by the strongly aversive effect of the offered noxiously cold plate.

The clinical effectiveness of a putative hypothermic agent may be enhanced by combining it with a conventional hypothermic strategy based on physical cooling [8]. Thus, we also determined if the hypothermic effect of carvacrol is potentiated by low ambient temperature. Interestingly, the hypothermic effect of carvacrol was abolished during physical cooling at 10°C in the current study. We propose the following interpretation of this finding. The hypothermic effect of carvacrol seen at typical room ambient temperature (24°C) suggests that carvacrol suppresses the basal level of heat generation. However, exposure to cold ambient temperature of 10°C triggers the potent compensatory cold-defense responses, including shivering and brown adipose tissue thermogenesis, which result in significant increase in heat generation. The absence of the carvacrol effect in these conditions suggests that it does not suppress these compensatory responses. The unexpected differential effect of TRPV3 activation on basal and stimulated levels of heat production is an interesting target for future research.

The main limitation of the current study is that it is unclear if the effects of carvacrol are fully mediated by the TRPV3 channels. Although carvacrol is known to be a potent activator of TRPV3 channels in vitro [18,22], it may also act on other receptors, such as TRPA1 and TRPM7 [23]. Thus, it is important to confirm that the effects of carvacrol described herein are indeed mediated specifically by TRPV3, possibly by repeating the study in TRPV3 knock-out mice and observing the absence of the effects found in wild-type mice.

## Conclusions

This study has demonstrated for the first time that systemic administration of the TRPV3 agonist carvacrol induces hypothermia in conscious mice, at least in part by decreasing whole-body heat generation. However, the substantially less pronounced hypothermic effect in rats, as well as significant adverse effects on blood pressure and survival at higher intravenous doses argue against the safe and specific actions of carvacrol on the thermoregulatory system. Thus, the TRPV3 activation by carvacrol appears unlikely to have a potential as a novel stand-alone pharmacological strategy for direct hypothermia induction in conscious human patients. However, because of the demonstrated effect of carvacrol on core temperature and blood pressure, further research into the role of the TRPV3 channels in controlling these variables may be useful for better fundamental understanding of the mammalian thermoregulation and other homeostatic autonomic responses.
